# Genetic analysis of a white-*to*-red berry skin color reversion and its transcriptomic and metabolic consequences in grapevine (*Vitis vinifera* cv. ‘Moscatel Galego’)

**DOI:** 10.1186/s12864-019-6237-5

**Published:** 2019-12-09

**Authors:** Vanessa Ferreira, José Tomás Matus, Olinda Pinto-Carnide, David Carrasco, Rosa Arroyo-García, Isaura Castro

**Affiliations:** 1 Centre for the Research and Technology of Agro-Environmental and Biological Sciences (CITAB), University of Trás-os-Montes and Alto Douro, 5000-801 Vila Real, Portugal; 2 Centre for Plant Biotechnology and Genomics (UPM-INIA, CBGP), Campus de Montegancedo. Autovía M40 km38, 28223 Pozuelo de Alarcón, Madrid, Spain; 3 Institute for Integrative Systems Biology, I2SysBio (Universitat de Valencia - CSIC), 46908 Paterna, Valencia Spain

**Keywords:** Grapevine, Berry color, Somatic variation, RNA-Seq, Moscatel Galego

## Abstract

**Background:**

Somatic mutations occurring within meristems of vegetative propagation material have had a major role in increasing the genetic diversity of the domesticated grapevine (*Vitis vinifera* subsp. *vinifera*). The most well studied somatic variation in this species is the one affecting fruit pigmentation, leading to a plethora of different berry skin colors. Color depletion and reversion are often observed in the field. In this study we analyzed the origin of a novel white-*to*-red skin color reversion and studied its possible metabolic and transcriptomic consequences on cv. ‘Muscat à Petits Grains Blancs’ (synonym cv. ‘Moscatel Galego Branco’), a member of the large family of Muscats.

**Results:**

The mild red-skinned variant (cv. ‘Muscat à Petits Grains Rouge’, synonym cv. ‘Moscatel Galego Roxo’), characterized by a preferential accumulation of di-hydroxylated anthocyanins, showed in heterozygosis a partially-excised *Gret1* retrotransposon in the promoter region of the *MYBA1* anthocyanin regulator, while *MYBA2* was still in homozygosis for its non-functional allele. Through metabolic (anthocyanin, resveratrol and piceid quantifications) and transcriptomic (RNA-Seq) analyses, we show that within a near-isogenic background, the transcriptomic consequences of color reversion are largely associated to diminished light/UV-B responses probably as a consequence of the augment of metabolic sunscreens (i.e. anthocyanins).

**Conclusions:**

We propose that the reduced activity of the flavonoid tri-hydroxylated sub-branch and decreased anthocyanin synthesis and modification (e.g. methylation and acylation) are the potential causes for the mild red-skinned coloration in the pigmented revertant. The observed positive relation between anthocyanins and stilbenes could be attributable to an increased influx of phenylpropanoid intermediaries due to the replenished activity of *MYBA1*, an effect yet to be demonstrated in other somatic variants.

## Background

The grapevine is one of the oldest perennial domesticated fruit crops in the world and it has been widely cultivated and valued either for its fruit or wine. Cultivation of domesticated grape (*Vitis vinifera subsp. vinifera*) started 6000–8000 years ago from its wild ancestor *V. vinifera* subsp. *sylvestris* in the Near East [1]. The large number of grape varieties known nowadays is certainly the result of many different processes, including multiple domestication centers from local *Vitis sylvestris* vines [2], subsequent crosses, and to a lesser extension, the conventional breeding practiced during the last century.

Vegetative propagation has been widely used as a strategy within breeding programs for multiplication of plants with desired features, creating clones that are genetically identical to the original donor. However, somatic mutations, naturally occurring during plant growth, can accumulate over time and generate divergent genotypes and occasionally lead to morphological and agronomical differences. These new interesting phenotypes can stabilize in grapevine plants as periclinal chimeras or extend to all cell layers, giving rise to new cultivars, in a process referred as clonal variation [3]. Consequently, somatic mutations combined with vegetative propagation have had a major role in increasing the genetic diversity in grapevine accessions. The use of these mutants in genomic studies is continuously helping to assign functions and roles to specific genes [3–6].

There are many examples of spontaneous variant traits, including berry color or flavor, ripening date, size and compactness of bunches, canopy growth or yield [5]. Vine growers have been exploring them as a source of diversity for both wine and table grapes. Genetic alterations responsible for these emergent phenotypes result from single nucleotide variation (SNV), insertion-deletions (INDELs) and from chromosomal rearrangements due to complex genome structural variation (SV) [4, 7]. The most well studied polymorphisms leading to somatic variations within grapevine varieties are those that affect berry skin pigmentation. Diversity in fruit color has led to a substantial classification of grape cultivars and wine classes in the market, a process that gained cultural significance and extends thousands of years into human history [8]. Grape skin color shows a great diversity of colors ranging from white or green to grey, pink, red and black. This color palette is determined by the differential accumulation of anthocyanins, a group of flavonoids, in epidermal and sub-epidermal cell layers of the berry skin.

The regulation of anthocyanin synthesis is directly related with the activity of several myeloblastosis-like (R2R3-MYB) transcription factors [9], some of which are located in two well-described grape color loci. The recently identified ‘vegetative color locus’ [10] harbors *VvMYBA5/6* and *VvMYBA7* genes, while the ‘berry color locus’ comprises *VvMYBA1* and *VvMYBA2* genes [11], two essential genes that determine berry skin color variation (Fournier-Level et al., 2009). Both loci share the regulation of late biosynthetic and modification/ transport-related genes, such as Uridine diphosphate (UDP)-glucose: flavonoid 3-*O*-glucosyltransferase (UFGT) and anthocyanin 3-*O*-glucoside-6″-*O*-acyltransferase (3AT) [10–12]. However, they differ in regulating the expression of the flavonoid-3′5’-hydroxylase (F3’5’H) family, directly influencing the proportion of tri and di-substituted anthocyanins [10], ultimately affecting color characteristics in terms of hues, values and saturations.

Mutations in *MYBA1* and *MYBA2* genes can cause a loss of transcription factor activity on anthocyanin biosynthetic genes, leading to a ‘white’ phenotype. The loss of berry skin pigmentation has been mostly associated with the insertion of the grape retrotransposon 1 (*Gret1*) retrotransposon in the 5′ regulatory region of the *MYBA1* gene [13]. Additionally, two mutations in the coding sequence of *MYBA2* (a point mutation and a 2 base pair (bp) CA deletion that alters its reading frame) can also contribute to the loss of berry skin pigmentation [11]. These altered gene structures are commonly designated as non-functional alleles (*VvmybA1a* and *VvmybA2w*, respectively), being frequently present in homozygosis in white-skinned cultivars [8, 14].

Several types of mutations have been identified at the berry color locus being responsible for color changes. Occasionally, black-skinned cultivars that are heterozygous for the non-functional and functional alleles give rise to color bud sports, characterized by red, grey or white-skinned berries depending on whether the mutations at the berry color locus occurred only in the L1 or both L1 and L2 cell layers [15–18]. Large deletions removing both functional *MYBA1* and *MYBA2* alleles have also been associated with color reversions from the black-skinned cultivars cv. ‘Cabernet Sauvignon’ and cv. ‘Pinot Noir’ to their white-skinned bud sports, cv. ‘Shalistin’ and cv. ‘Pinot Blanc’, respectively [18, 19]. Moreover, in cv. ‘Koshu’, a weakly colored grape cultivar, a 33 bp insertion in the second intron of the *MYBA1* red allele affects messenger RNA (mRNA) stability [20]. More recently, Carbonell-Bejerano et al. [7] demonstrated that the loss of color in cv. ‘Tempranillo Blanco’, occurs in response to an unbalanced chromoanagenesis, a process in which a large number of complex rearrangements occur in a single catastrophic event, as often observed in cancer cells [21].

On rare occasions, reversions from mutated-to-functional allelic versions may occur in white-skinned cultivars giving rise to red-skinned variants. The main mechanism described for color gain is the partial *Gret1* retrotransposon excision from the *VvMybA1* promoter, leaving behind its solo-3′ long terminal repeat (LTR) region (*VvmybA1b* allele). This mechanism has been firstly described in cv. ‘Ruby Okuyama’ and cv. ‘Flame Muscat’ by Kobayashi et al. [13] but has been associated with several other red-skinned somatic variants derived from white-skinned cultivars [16]. In addition, the pink-skinned somatic variant cv. ‘Benitaka’ derived from the white-skinned cv ‘Italia’ was reported as a result of homologous recombination between the non-functional allele of *MybA1* and the truncated *MybA3* gene at their promoter region, resulting in the recovery of *MybA1* genomic integrity (and therefore its transcription) on cv. ‘Benitaka’ [22].

Skin color reversion is a rather common event in grapevine and currently, several pigmented and unpigmented varieties have certified clones with different skin tints. ‘The Muscat à Petits Grains Blancs’ cultivar (synonym cv. ‘Moscato Bianco’, cv. ‘Moscatel de Grano Menudo’, or cv. ‘Moscatel Galego Branco’ as it is known in Portugal) is considered one of the main progenitors of the large family of Muscats, extensively spread all over the world and appreciated since ancient times mainly due to its highly terpenic flavor [23]. Historically, the appearance of the ampelographic reference to cv. ‘Moscato Rosso’ perfectly resembling the already known cv. ‘Moscato Bianco’ [24] suggested that the red-skinned variant derived from the white-skinned cultivar, probably as the result of a selection episode in a cv. ‘Moscato Bianco’ vine. Although different color variants with red shades are known, a previous study analyzing three accessions of ‘Moscatel Galego’ with different color shades (white, red and black) revealed that only the white and red-skinned accessions have the same Simple Sequence Repeat (SSR) profile, suggesting that the black-skinned accession was a different variety [25].

For the Muscats family, many pink and red berry color variants are commonly known (http://plantgrape.plantnet-project.org/). In this study we analyzed the genetic origin of a white-*to*-red skin color reversion on a color somatic variant of cv. ‘Moscatel Galego Branco’. In addition, through metabolic and transcriptomic (RNA-Seq) analyses we studied the possible consequences of pigment depletion and reversion.

## Results and discussion

### Berry color phenotypes of cv. ‘Moscatel Galego’ variants

**Fig. 1:**
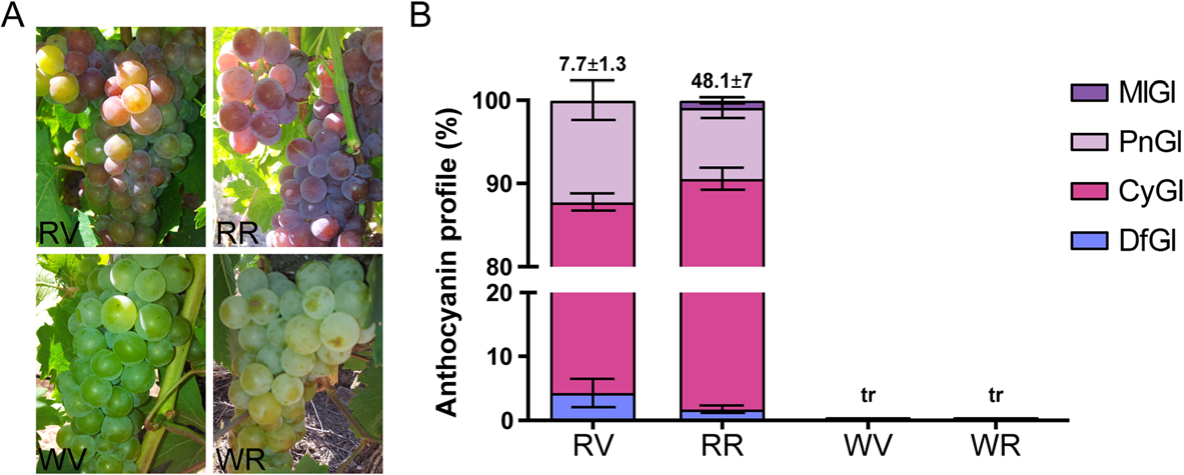
Color reversion in cv. ‘Moscatel Galego Roxo’ is mainly attributable to the regaining of di-hydroxylated anthocyanin accumulation. **a** Representative plant phenotypes in the field at veraison and ripening of cv. ‘Moscatel Galego Roxo’ and cv. ‘Moscatel Galego Branco’. **b** Anthocyanin quantifications. RV and RR: red-skinned variant at veraison and ripening, respectively. WV and WR: white-skinned variant at veraison and ripening, respectively

The different color phenotypes of ‘Moscatel Galego’ cultivars used in this study are shown in Fig. 1a, with cv. ‘Moscatel Galego Branco’ being a typical white-skinned cultivar, whereas its color-reverted variant cv. ‘Moscatel Galego Roxo’ shows a red blush coloration. In a previous study we measured different colorimetric parameters (a*, b*, L*, hue angle and chromaticity) during berry development, in order to investigate the differences between the two skin color phenotypes of cv. ‘Moscatel Galego’ [26]. Here, we observed an inverse correlation between a* and b* values with anthocyanin accumulation (and therefore ripening) in cv. ‘Moscatel Galego Roxo’; the strong correlation with a* agreeing with a red (and less blueish) color trait [26]. Moscatel Galego variants differ in pigmentation with cv. ‘Moscatel Galego Tinto’, a black-skinned cultivar with a different Simple Sequence Repeat (SSR) molecular marker profile [25]. In this previous work, we also showed that red-skinned cv. ‘Moscatel Galego Roxo’ skin possessed less anthocyanins than other black cultivars grown in the area (e.g. Pinot Noir), and even 10 times less anthocyanins than Pinot Gris [25]. Taken altogether, we propose that the pigmented revertant of cv. ‘Moscatel Galego’ has ‘mild-red’ skin.

### Di-hydroxylated anthocyanin derivatives are the most abundant in cv. ‘Moscatel Galego Roxo’

We quantified anthocyanin derivative compounds in both Muscat variants at two developmental stages: veraison and ripening (2 weeks after veraison - WAV). As expected, anthocyanins were only detected in cv. ‘Moscatel Galego Roxo’. Only four anthocyanins were identified (all being monoglucoside derivatives): delphinidin, cyanidin, peonidin and malvidin (Fig. 1b). At veraison and ripening stages, cyanidin-3-*O-*glucoside accounted for 83.46 and 88.80% of the total amount of anthocyanins, respectively. The next most abundant anthocyanin was peonidin-3-*O*-glucoside, accounting for 12.24% at veraison and 8.57% at ripening. The tri-hydroxylated anthocyanin derivatives represented the less abundant anthocyanins, where delphinidin-3-*O*-glucoside showed 4.2 and 1.7% at veraison and ripening stage, respectively, while malvidin-3-*O*-glucoside was only detected at the ripening stage (0.86%). These results are in agreement with Ferreira et al. [25], where in fully mature berries the di-hydroxylated cyanidin-3-*O-*glucoside was the most abundant. Also, at this period, a fifth minor anthocyanin corresponding to petunidin-3-*O-*glucoside was found suggesting its accumulation and ripening stages later in development [25].

### The color reversion in cv. ‘Moscatel Galego Roxo’ is influenced by *Gret1* retrotransposon partial excision from *MYBA1* promoter and is not due to a recovery of the functional *MYBA2* allele

Twelve SSR markers, including the six SSR markers proposed by This et al. [27] and adopted by the *Organisation Internationale de la Vigne et du Vin* [28] for varietal identification, were used to genotype the white-skinned cv. ‘Moscatel Galego Branco’ and its color reverted variant cv. ‘Moscatel Galego Roxo’ in order to ascertain the genetic identity between them. In fact, this fingerprinting system showed an exact match between cv. ‘Moscatel Galego Branco’ and cv. ‘Moscatel Galego Roxo’ allelic profiles for all the 12 SSR loci analyzed, confirming that both cultivars are very closely related and probably they were originated recently from each other (Table 1). This genetic identity was further confirmed by comparison with the fingerprinting reported in previous works [25, 29].

**Table 1:** Genetic profiles of cv. ‘Moscatel Galego Branco’ and its red-skinned revertant variant cv. ‘Moscatel Galego Roxo’ based on a set of microsatellite markers used for true-to-type confirmation (12 SSR loci) and for characterization of the berry color locus and its surrounding genomic region (10 SSR loci). The grey background indicates the putatively homozygous regions. ho – homozygous; Gret1 – non-functional allele; Solo3’LTR – functional allele

		LG^a^	11	16	7	5	7	5	12	3	4	19	10	15
			VVS2	VVMD5	VVMD7	VVMD27	VrZAG62	vrZAG79	VMC4f3	VVMD28	VVMD32	VVIp31	VVIv37	VVIv67
Cultivar	Layer	Berry skin Color^b^												
Moscatel Galego Roxo	L1 + L2	R	130–130	224–232	231–247	175–191	185–195	249–253	165–204	243–265	261–269	182–186	159–161	360–371
Moscatel Galego Branco	L1 + L2	W	130–130	224–232	231–247	175–191	185–195	249–253	165–204	243–265	261–269	182–186	159–161	360–371
		LG^a^	2											
			SC8_010	SC8_026	VVNTM1	VVNTM2	VvMYBA2R44	VvMYBA1	VVNTM3	VVNTM5	VVNTM6	VVNTM4	VVIU20	VMC7G3
			12,674	12,970	14,149	14,151	14,181	14,248	14,288	14,325	14,330	14,384	16,539	18,270
Cultivar	Layer	Berry skin Color^b^												
Moscatel Galego Roxo	L1 + L2	R	ho	ho	ho	ho	T/T	Gret1/Solo 3’LTR	ho	ho	ho	ho	ho	ho
	L2		ho	ho	ho	ho	T/T	Gret1/Solo 3’LTR	ho	ho	ho	ho	ho	ho
Moscatel Galego Branco	L1 + L2	W	ho	ho	ho	ho	T/T	Gret1	ho	ho	ho	ho	ho	ho
	L2		ho	ho	ho	ho	T/T	Gret1	ho	ho	ho	ho	ho	ho

^a^ LG – Linkage group; ^b^ R – Red; W - White

To further investigate the genetic structure of the berry color locus and its surrounding genomic region, 12 molecular markers were screened, 10 SSRs spread throughout this region of chromosome 2 and two R2R3-MYB genes, *VvMYBA1* and *VvMYBA2*, which were analyzed regarding their functional and non-functional allelic configurations (Table 1). This investigation was performed by using a well-established layer-specific approach, which has already been proven to be a successful method to decipher the molecular mechanisms responsible for color reversions on other grape somatic variants [15, 16, 30].

**Fig. 2:**
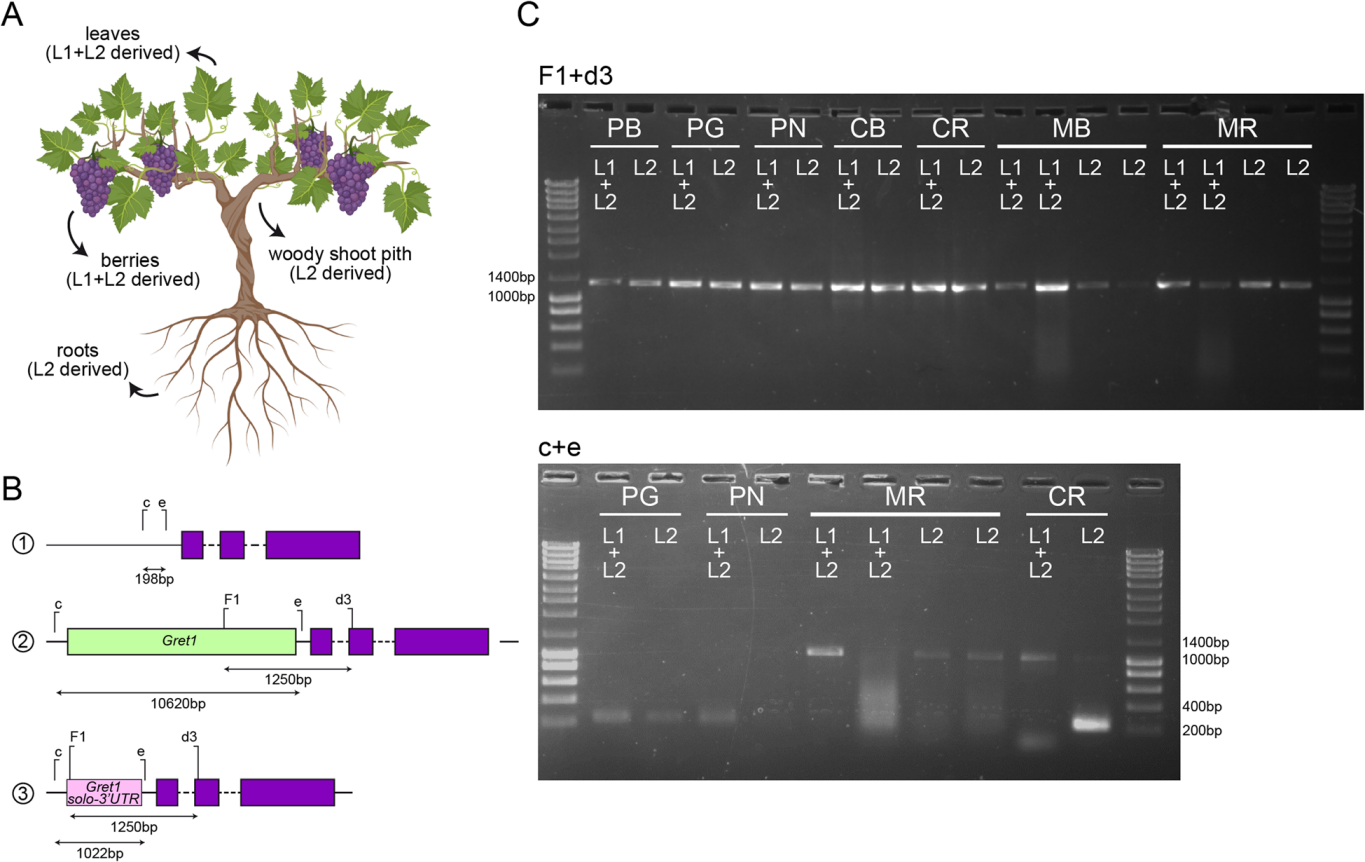
Molecular marker analysis reveals the genetic nature of color reversion in cv. ‘Moscatel Galego’ variants. **a** Cartoon depicting the different plant tissues and respective cell layers used for molecular analyses; **b** Schematic representation of *MYBA1* alleles (1- *VvmybA1c*; 2 – *VvmybA1a*; 3 – *VvmybA1b*) and genomic location of the primer combinations used in the PCR assays [F1 + d3 – VvMYBA1(1) and c + e – VvMYBA1(2)]; **c** PCR assays performed on cv. ‘Pinot Blanc’ (PB), cv. ‘Pinot Gris’ (PG), cv. ‘Pinot Noir’ (PN), cv. ‘Chasselas Blanc’ (CB), cv. ‘Chasselas Roxo’ (CR), cv. Moscatel Galego Branco’ (MB) and cv. ‘Moscatel Galego Roxo’ (MR) using VvMYBA1(1) (1250 bp – *VvmybA1a*) and VvMYBA1(2) (198 bp – *VvmybA1c* and 1022 bp – *VvmybA1b*) primer combinations. This figure was created using BioRender.com

Different assays were performed to characterize the *VvMYBA1* locus; the first one [VvMYBA1(1)] aimed to investigate the insertion of the *Gret1* retrotransposon at the gene promoter region (*VvmybA1a* allele), which was detected in both white and red-skinned variants of the cv. ‘Moscatel Galego’ in both L1 + L2- and L2-derived tissues (Fig. 2a- c). The second assay [VvMYBA1(2)] was carried out to identify the wild-type allele (*VvmybA1c* allele) or other potential functional alleles, such as the *Gret1* partial excision [solo-3′ LTR allele, also known as *VvmybA1b* allele], which was detected for cv. ‘Moscatel Galego Roxo’, also in both L1 + L2- and L2-derived tissues (Fig. 2a- c).

Our analysis showed a clear amplification of the *Gret1* allele (non-functional) (Fig. 2 panel C, F1 + d3 primer combination) and despite not shown, a PCR with the c + e primer combination was indeed performed on cv. ‘Moscatel Galego Branco’ determining that the white-skinned variant has a complete absence of functional *MYBA1* alleles. These results suggests that cv. ‘Moscatel Galego Branco’ is homozygous for the presence of the *Gret1* allele in both L1 and L2 cell layers. The results obtained for cv. ‘Moscatel Galego Roxo’ showed that this cultivar is heterozygous for the presence of the *Gret1* and the solo-3′ LTR alleles in both the L1 + L2 (leaves and berry skin) and L2 layer-derived (roots and pith wood) tissues (Fig. 2a- c), as it has been described for cv. ‘Moscato Bianco’ and ‘Moscato Rosso’ by Migliaro et al. [16] and also for other red-skinned cultivars derived from a white-skinned ancestor, such as cv. ‘Chasselas Rouge’, cv. ‘Italia Rubi’, cv. ‘Malvasia Rosa’, and cv. ‘Sultanina Rosa’. Despite the presence of the functional allele solo-3′ LTR is not clearly observed in the L1 + L2-derived from berry skins of the cv.‘Moscatel Galego Roxo) due to the low quality of the extracted gDNA, the amplification from young leaves confirms that the red-skinned variant cv. ‘Moscatel Galego Roxo’ has a functional allele (solo-3’ LTR) (Fig. 2c). Similarly, the ‘positive control’ cv. ‘Chasselas Roxo’ possesses the solo-3′ LTR allele in both L1 + L2 and L2-derived tissues (1022 bp band, Fig. 2c), as well as the presence of an unspecific amplification of MYBA2 on the L2-derived tissue (~ 250 bp), as described by Migliaro et al. [16]. Consequently, it can be hypothesized that the partial excision of the *Gret1* retrotransposon, leaving the solo-3′ LTR region, must have occurred at least in the L2 cells of the homozygous ancestor cv. ‘Moscatel Galego Branco’, giving rise to a red-skinned somatic variant. This hypothesis agrees with the historical background of ‘Moscatel Galego’ variety and has also been described as the main mechanism for color recovery on white-skinned cultivars [16, 24].

Similarly to what has been previously observed in other studies for white-skinned ancestor cultivars [16, 30], an extensive putatively homozygous and monomorphic region was found along the distal arm of chromosome 2, including the presence of the non-functional T allele of *VvMYBA2* in homozygosis both in cv. ‘Moscatel Galego Branco’ and ‘Moscatel Galego Roxo’ (Table 1). Altogether we suggest that the non-black, but mild red skin coloration is recovered from the white phenotype by a partial *MYBA* activation (i.e. excluding *MYBA2* gain of function), occurring at least in the L2 cell layer. As a chimeric state of the reverted allele (i.e. partial MYBA1 activation occurring only in the L2) is unlikely (or at least cannot be implied with our data), it is possible that the presence of the non-excised *Gret1* LTR region in the promoter of the red revertant allele may have a negative effect on *MYBA1* gene transcription, probably by interfering with the binding of transcription factors on regulatory elements present in the promoter or 5′ untranslated region (5’UTR).

### Transcriptomic comparison of color variants reveals a specific modulation of light responsive genes and secondary metabolism

Since both cv. ‘Moscatel Galego’ variants represent near-isogenic lines one from the other, we decided to explore the transcriptomic differences caused by their color variation. mRNA libraries were constructed for the four previously tested samples: red and white, at veraison and ripening (RV, WR, RR and WV) and pair-ended sequenced by Illumina (three biological replicates per condition). After adaptor and low-quality base trimming, 952,357,192 clean reads (39.68 million reads in average per condition) remained. An average of 88.7% of reads/condition mapped uniquely to the reference genome, while 4.5% mapped to multiple loci and were discarded. Principal component analysis (PCA) showed that a majority of the variation in abundances of mRNAs between libraries is associated with developmental stage [Principal Component 1 (PC1) of 69.2%; (Additional file 1 A)], while PC2 was inferred to capture predominantly somatic variant variation (24.5%). The differential expression analysis, run through DESeq2, showed 2551 and 2785 genes to be up- and down-regulated [False discovery rate (FDR) < 0.05] by color reversion at veraison, compared with 4275 and 4223, occurring at ripening, respectively (Additional file 1 B). This indicates that the biggest differences between cv. ‘Moscatel Galego Roxo’ and cv. ‘Moscatel Galego Branco’, in terms of the number of differentially expressed genes (DEGs), are found after the onset of ripening [expression measures in Fragments per kilobase of transcript per million mapped fragments (FPKM) values of around 30 K genes is found in (Additional file 2)].

We analyzed the proportion of enriched gene ontology (GO) categories in the color reverting variant and found that different environmental, metabolic and stress responses were enriched (Additional files 3, 4 and 5). As expected, flavonoid metabolism was enriched in the up-regulated genes, as reflected by many different categories such as ‘chalcone isomerase activity’, ‘phenylalanine ammonia-lyase activity’ and ‘phenylpropanoid biosynthetic process’. Interestingly, among up-regulated genes found both at veraison and ripening, there is an enrichment of ‘anaerobic respiration’ (GO:0009061), ‘cutin biosynthetic process’ (GO:0010143), ‘trihydroxystilbene synthase activity’ (GO:0050350) and ‘response to heat’ (GO:0009408) terms. Veraison-specific highly enriched biological processes included ‘production of small interference RNA (siRNA) involved in RNA interference’ (GO:0030422, FDR = 0.008) and ‘histone acetyltransferase activity’ (GO:0004402, FDR = 0.02), while at ripening the terms ‘regulation of auxin mediated signaling pathway’ (GO:0010928, FDR = 0), ‘trehalose metabolism in response to stress’ (GO:0070413, FDR = 0.01), ‘xyloglucan biosynthetic process’ (GO:0009969, FDR = 0.02) and ‘cell wall biogenesis’ (GO:0042546, FDR = 0.02) were enriched.

Enriched GO categories of down-regulated genes in response to color reversion showed three major processes occurring with higher rank in the white-skinned variant: photosynthesis, light responses and isoprenoid metabolism. Within the former, at least 20 related terms were enriched at both stages, harboring photosystem components, chloroplast structures and chlorophyll synthesis. Several light-signaling categories were enriched at both developmental stages including ‘response to high light intensity’ (GO:0009644) and ‘response to low fluence blue light stimulus by blue low-fluence system’ (GO:0010244). Metabolic responses to be down-regulated with color reversion were mainly subscribed to the metabolism of lipids (e.g. GO:0030148, GO:0006633, GO:0008610), steroids (GO:0006694 and GO:0006696), farnesyl-diphosphate (GO:0004310), squalene (GO:0051996), xanthophylls (GO:0016123) and carotenoids (GO:0016117). These results suggest that color reversion arrests photosynthesis and the accumulation of accessory pigments as a response to sunlight filtering, mainly exerted by anthocyanins. In contrast to the ‘heat response’ term identified among genes induced by color reversion, we found the term ‘response to cold’ (GO:0009409) enriched in down-regulated genes at both developmental stages, suggesting that white- and red-skinned berries may have different daytime temperatures possibly due to the physico-chemical properties of pigments in sunlight reflection and absorption. Additionally, phosphate ion transport-related terms (e.g. GO:0035435 and GO:0006817) were also enriched among down-regulated genes at both developmental stages.

### Inactivation of the flavonoid tri-hydroxylated sub-branch and decreased anthocyanin synthesis and modification reactions as potential causes for the mild red-skinned coloration in cv. ‘Moscatel Galego Roxo’

**Fig. 3:**
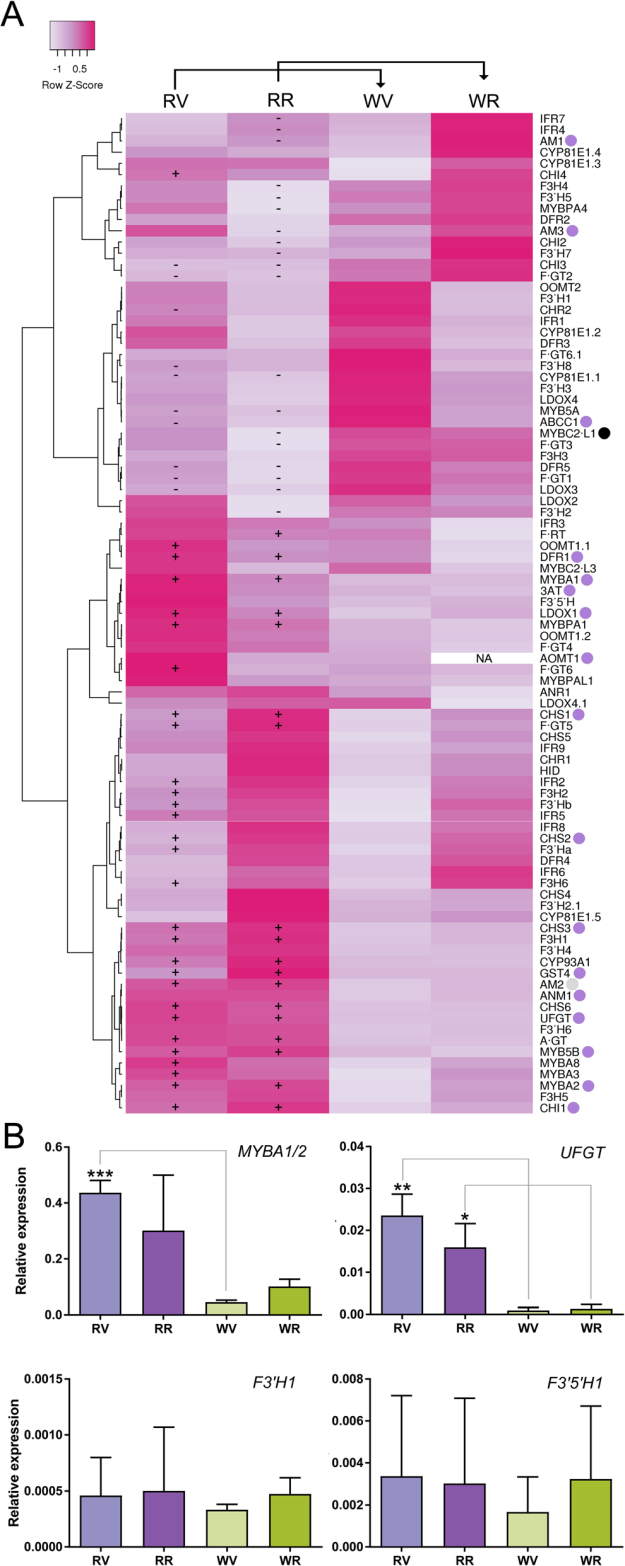
Expression behavior of phenylpropanoid and anthocyanin related genes in color reverted somatic variants. **a** Expression heatmap for early and late phenylpropanoid pathway genes. Genes with significant expression differences between the color reverting and the white-skinned cultivar (as reference) are shown with ‘+’ and ‘-’ symbols representing up and down-regulation, respectively (FDR < 0.05). Purple dots next to gene names represent those with proven roles in anthocyanin synthesis. The anthocyanin-specific repressor MYBC2-L1 (subgroup 4) is highlighted with a black dot. Negative and positive Z-scored FPKM values represent low and highly expressed genes, respectively, while values around zero correspond to mild expression levels. Average linkage clustering and Pearson distance measurement methods were used for clusterization. NA = not assigned value due to very low expression. Gene descriptions, together with their FPKM values can be found in Additional file 6. **b** qPCR validation of selected genes (**c**) qPCR expression analysis of selected genes: MYBA1, its target UFGT and the flavonoid hydroxylases F3’H (VIT_17s0000g07210) and F3’5’H (VIT_06s0009g02840). One, two and three asterisks represent significant differences of *p* < 0.1, *p* < 0.05 and *p* < 0.01, respectively in the red to white variant comparisons independently on each developmental stage (unpaired t-test, two-tailed)

We further inspected the expression of early-phenylpropanoid and anthocyanin-related genes between both color variants to corroborate the metabolic data and gene ontology analysis (Additional file 6). Phenylalanine ammonia lyase (PAL), cinnamic acid 4-hydroxylase (C4H) and 4-coumarate: CoA ligase (4CL), being key enzymes catalyzing the first three steps of the phenylpropanoid pathway (PP), had many transcripts being up-regulated on cv. ‘Moscatel Galego Roxo’, particularly at the berry ripening stage (Fig. 3a). This higher expression levels suggest an increased influx of the PP pathway, ultimately affecting down-stream pathways.

From all the genes of the PP shown in Fig. 3a-b, the most affected in the color reverting condition were those related to anthocyanin synthesis (defined by purple dots). This increase on anthocyanin-related genes (e.g. UFGT – VIT_16s0039g02230, the last committed step for anthocyanin synthesis) in cv. ‘Moscatel Galego Roxo’ coincides with the accumulation of anthocyanins in the red-skinned variant. The transcript expression pattern of the major anthocyanin regulators MYBA1, MYBA2 and MYB5B completely matches with the expression of their target genes (such as UFGT [31], 3AT [12], GST4 and AOMT1) on cv. ‘Moscatel Galego Roxo’. *MYBA1* expression also agrees with our genetic data (merely no detection on white berries, < 0.5 FPKM) corroborating with the allelic composition of the *MYBA1* gene in both variants. *MYBA2* transcripts were highly detected in white-skinned berries (70–130 FPKM) although the highest levels were found in the red variant. Either way, these expression patterns should not be relevant once our genetic data showed that both color variants are homozygous for the non-functional T alleles. We further validated the molecular marker data by inspecting all reads mapping at the *MYBA2* locus. Both the G-*to*-T single nucleotide polymorphism at position 131 of the CDS (that leads to a R^44^➔L^44^ amino acid substitution), and the CA dinucleotide deletion in Exon 3 (disrupting the C-terminal) were found in all reads belonging to veraison and ripening samples of both somatic variants. These two mutations are responsible, according to Walker et al. [11], for MYBA2’s inactivity in the white allele.

In order to search further proofs of potential causes for the mild red-skinned coloration in cv. ‘Moscatel Galego Roxo’ we integrated an additional RNA-Seq analysis of a purple-*to*-red color somatic variation found in the table grape cv. ‘Red Globe’ (SRA BioProject PRJNA539972). This analysis allowed us to see that all last biosynthetic steps of anthocyanin synthesis (including anthocyanin modification steps, i.e. *UFGT*, *GST4*, *AOMT* and *3AT* genes) had null expression in the white Muscat and were lowly expressed in the red Muscat variant when compared to the red and purple variants of cv. ‘Red Globe’ (Additional file 7 A). In fact, a clear and gradual transition from cv. ‘MGB’ to ‘MGR’ to ‘Chimenti Globe’ to ‘Red Globe’ explained most of the variability in the anthocyanin-gene expression data (Additional file 7 B).

Flavonoid 3′-hydroxylases (F3’H) and 3′5′-hydroxylases (F3’5’H) are the enzymes that catalyze the hydroxylation of the B-ring of flavonoids, producing the corresponding di-hydroxylated and tri-hydroxylated derivatives, respectively (i.e. found in both flavonol and anthocyanin compounds). In grapevine, the variation in anthocyanin composition is strongly influenced by the expression of genes coding for flavonoid hydroxylases [32–34]. Usually F3’5’H activity prevails over F3’H, and the products of flavonoid hydroxylases are predominately channeled into the branch of the pathway involved in the biosynthesis of delphinidin (which is latter transformed into malvidin, all with blue-purplish coloration) at the expense of those involved in the synthesis of cyanidin (reddish derivatives). Jeong et al. [32] suggested that the levels of F3’Hs and F3’5’H expression agreed well with the ratios of cyanidin- and delphinidin-based anthocyanins, which is in accordance with Castellarin et al. [33, 34], who found a strong relationship between the expression of *VvF3’H* and *VvF3’5’H* genes and the kinetics of accumulation of di-hydroxylated and tri-hydroxylated anthocyanins in the dark blue-skinned cv. ‘Merlot’. In the current study, transcripts coding for F3’5’H were relatively lowly abundant and expressed without many differences between both color variants. In addition, a higher expression of a few F3’H transcripts was observed, but with no differences between red and white-skinned berries.

**Fig. 4:**
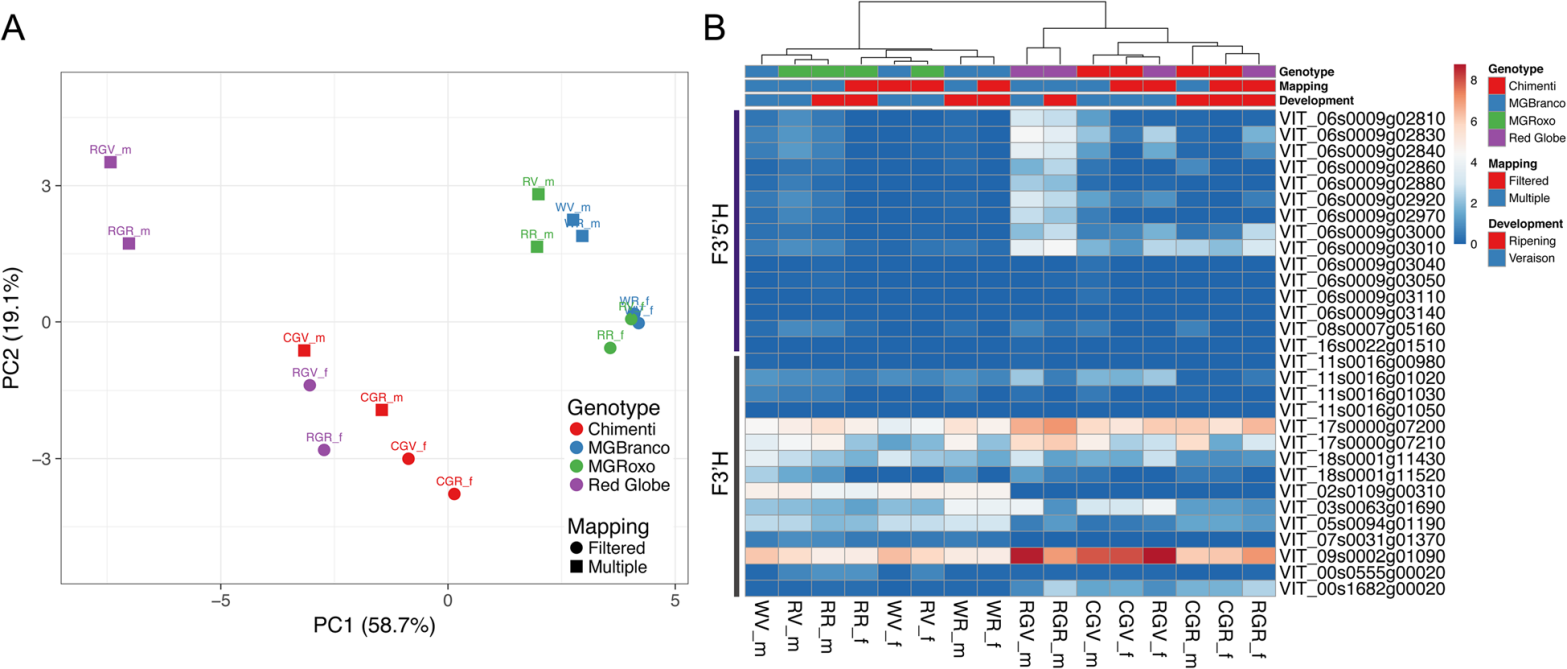
Effects of filtering multi-mapped reads in the calculation of gene expression of the flavonoid 3′ and 3′,5′ hydroxylase gene families in different somatic color variants. The cv. ‘Moscatel Galego’ experiment was coupled to RNA-Seq data generated in cv. ‘Red Globe’ (RG) and its less-pigmented color variant cv. ‘Chimenti Globe’ (CG; Santibañez et al., 2019). **a** PCA plot showing the effect of filtering, produced from logFPKM+ 1 values of both hydroxylase gene families. Clustering of samples according to genotype, developmental stage and read mapping procedure is shown. **b** Heatmap of logFPKM+ 1 transformed data comparing multi-mapped read filtering (f) and non-filtering (m). Samples (columns) are clustered using correlation distance and average linkage

Most F3’5’H family genes are located in chromosome (chr) 6, and with the exception of one gene located in chr 8, all arose from sequential tandem duplications [35]. Because of this, F3’5’H genes are extremely similar. In order to discard the effect of multiple-mapped reads filtering in the final calculation of *F3’5’H* gene expressions we calculated and compared FPKM values of each *F3’H* and *F3’5’H* gene with and without filtering and extended the analysis to the cv. ‘Red Globe’ and cv. ‘Chimenti Globe’ color somatic variants (Fig. 4). Despite there is a clear effect of filtering multi-mapping reads in the final gene expression (as seen in the PCA plot of Fig. 4a), the tendencies are maintained, with a very low expression in cv. ‘Moscatel Galego’ variants, a higher expression in cv. ‘Chimenti Globe’ and an even higher expression in cv. ‘Red Globe’ (Fig. 4b).

These data suggest that flavonoids in both variants are majorly channeled into the di-hydroxylated branch due to the activity of the F3’Hs VIT_17s0000g07200 and VIT_09s0002g01090. In the case of the red-skinned variant, these flavonoids are then transformed into anthocyanin compounds. The idea of a deficient activation of the tri-hydroxylated branch would correlate with the higher accumulation of cyanidin-3-*O*-glucoside (83.46% at veraison and 88.80% on ripe berries) in the downstream steps of this pathway in the red-skinned cv. ‘Moscatel Galego Roxo’ whereas tri-hydroxylated anthocyanins were only slightly accumulated (Fig. 1b).

In concordance with the results obtained from overexpressing MYBA1 in cv. ‘Chardonnay’ or silencing MYBA1 in cv. ‘Shiraz’ [12], Matus et al. [10] observed that after the overexpression of MYBA1 in grapevine hairy roots, most of the *F3’5’H* genes were activated. On the contrary, the closely related MYBA6 and MYBA7 were not able to activate the family, in correlation with higher di/tri-hydroxylated anthocyanin ratios. The differences between MYBA1/A2 and A6/A7 mostly relies on the presence of a C-terminal motif conferring high induction capacity of target genes, in addition to other residues with non-proven functionality yet. Despite the sequence of MYBA1 in cv. ‘Moscatel Galego Roxo’ is identical to that of other red and black-skinned cultivars, our data suggests that the inability of activating the tri-hydroxylated branch could respond to a lower MYBA1 dosage and a null MYBA2 function. In the case of MYBA1, we could only speculate that a smaller number of cells could be expressing this gene (i.e. those located exclusively in the L2 cell layer) in comparison to black-skinned berries (expressing high amounts of *MYBA1* and a functional *MYBA2* in both L1 and L2 cell layers).

The methylation of phenolic compounds, as catalyzed by *O*-methyltransferases (OMTs), is an important step in flavonoid metabolism [33]. In grapevine, increasing evidence suggests that the expression of *OMT* genes is correlated with the accumulation of methylated anthocyanins [36, 37], For instance, cyanidin and delphinidin are respectively converted to peonidin and malvidin by the action of 3′-*O-*methyltransferase. We determined that peonidin-3-*O*-glucoside was the second major anthocyanin in cv. ‘Moscatel Galego Roxo’ in correlation with the expression profile observed for *AOMT1* despite it was found in low levels. Additionally, the acylation step of anthocyanin biosynthesis is mediated by anthocyanin acyltransferases, which promotes color stability and intensity of anthocyanins [38]. In the present study acylated forms of anthocyanins were not detected, which agrees with the fact that the unique transcript detected 3AT (VIT_03s0017g00870) is expressed at very low levels in cv. ‘Moscatel Galego Roxo’.

Altogether, the RNA-Seq data generated in this study, coupled to the reanalysis of public available RNA-seq data derived from the study of a purple-*to*-red somatic color variation event, allows us to hypothesize that the mild red coloration in cv. ‘Moscatel Galego Roxo’ berries results from the poor activation of the flavonoid tri-hydroxylated sub-branch and decreased anthocyanin synthesis (due to low *UFGT* and *GST4* expressions), also including methylation/acylation activities due to even lower expressions of *3AT* and *AOMT* genes in the Muscat cultivars. This scenario, together with a complete inactivation of *MYBA2* could be responsible for the low activity of the anthocyanin branch in the mild-red skinned somatic variant cv. ‘Moscatel Galego Roxo’.

### Stilbenes differentially accumulate in red-skinned somatic variants through the increased expression of their regulators

Stilbenes constitute an important group of phenylpropanoids that share the first steps of the general phenylpropanoid pathway with flavonoids and have been extensively studied due to their nutraceutical properties and also because of their plant protective roles against pests, pathogens and abiotic stresses [39]. Since we observed enriched stilbene-related ontology terms in the red skinned somatic variant, we further evaluated the expression of the complete stilbene pathway, including their characterized regulators: the R2R3-MYB transcription factors MYB14 and MYB15 [40], as well as the recently reported candidate Tryptophan-arginine-lysine-tyrosine protein domain regulators (WRKY), WRKY3, WRKY24 and WRKY43 [39].

**Fig. 5:**
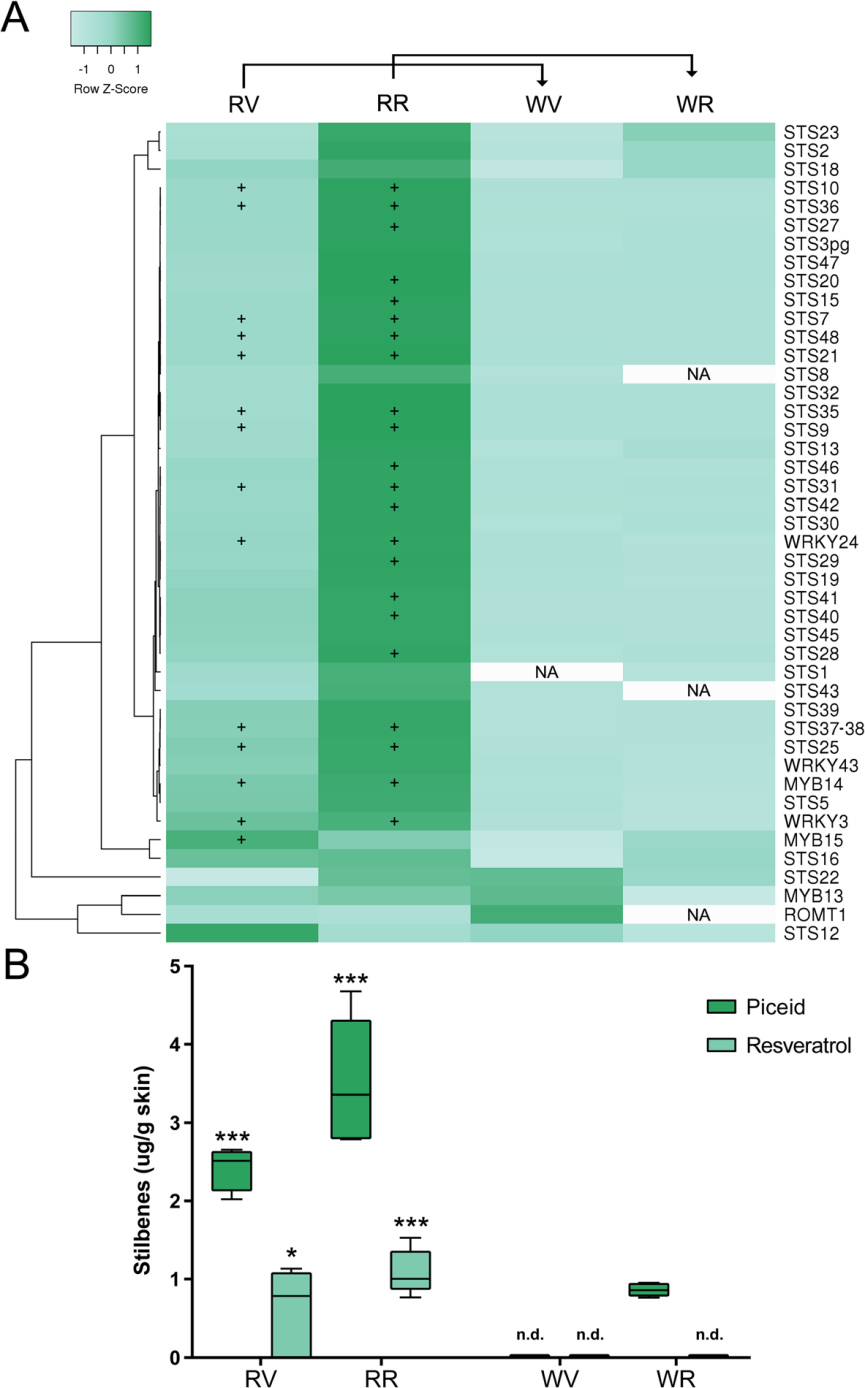
Activation of the stilbene pathway in the berry skin of the color reverting variant. **a** Expression changes of structural and regulatory genes of the stilbene pathway. Genes with significant expression changes between the color reverting and the white-skinned cultivar (as reference) are shown with the ‘+’ symbol representing up-regulation (FDR < 0.05). Average linkage clustering and Pearson distance measurement methods were used for clusterization. NA = not assigned value due to very low expression. Gene descriptions, together with their FPKM values can be found in Additional file 6. **b** Resveratrol and piceid quantifications in berry skins of cv. ‘Moscatel Galego’ variants. One, two and three asterisks represent significant differences of p < 0.1, p < 0.05 and p < 0.01, respectively in the red to white variant comparisons independently on each developmental stage (unpaired t-test, two-tailed). nd: not detected

The cv. ‘Moscatel Galego Roxo’ showed an increased expression of stilbene related genes compared to its white-skinned variant. At the ripening stage it presented a significant up-regulation of many *STILBENE SYNTHASE* (*STS*) genes (22 transcripts among 37) that were not induced in the white-skinned variant (Fig. 5a). Despite being less expressed when compared to ripening, 14 *STS* transcripts were up-regulated in the red-skinned variant at veraison. The transcript profiles are in accordance with the resveratrol and piceid contents, accumulating 5.3 times higher in the red variant at ripening (Fig. 5b). These results suggest, that despite the fact that the flavonoid and stilbene branches are competing with each other for p-coumaroyl-CoA and cinnamoyl-CoA substrates [41] (STS and chalcone synthases belong in fact to overlapping homologous enzyme superfamilies; Interpro IPR012328), an increased flux into the phenylpropanoid pathway may have an effect in the availability of stilbene precursors which in turn may activate the transcription of *STS* genes by positive feedback.

Since several transcriptional regulators of the stilbene biosynthetic pathway have been identified in grapevine (all of them showing a strong co-expression with *STS* genes) we hypothesized that the activation of *STS* genes in cv. ‘Moscatel Galego Roxo’ could be due to an increase in the expression of their regulators. The R2R3-MYB gene *MYB14* showed increased expression in the red-skinned variant at both developmental stages (Fig. 5a) although its expression was much higher at ripening, correlating with the significant higher red-*to*-white (R/W) expression ratio of its proven targets *STS29* (VIT_16s0100g01010) and *STS41* (VIT_16s0100g01130) [42] at this later stage. *MYB15* had a significantly increased R/W expression ratio only at veraison, suggesting that this MYB may have a more initial role in *STS* activation. Wong et al. [40] showed that *MYB13,* sharing a close similarity with *MYB14* and *MYB15* (all being part of Subgroup 2), was highly co-expressed with several *STS* transcripts. Despite our results in cv. ‘Moscatel Galego Roxo’ do not show such correlation this could be due to the fact that *MYB13* is more expressed in tissues other than fruits, such as roots and leaves.

Very recently, Vannozzi et al. [39] identified and characterized TFs from the WRKY TF family (*WRKY03*, *WRKY24*, *WRKY43* and *WRKY53*) as potential *STS* regulators. These authors observed that *WRKY24* (VIT_08s0058g00690) seems to act as a direct effector in the activation of the *STS29* promoter, independently of *MYB14* and *MYB15*. We observed that the expression levels of the *WRKY24* and *STS29* were coordinated in cv. ‘Moscatel Galego Roxo’. Vannozzi et al. [39] showed that *WRKY03* did not have an effect on *STS29* promoter activity on its own, but acted in combination with *MYB14*. These two WRKY TFs were differently increased in cv. ‘Moscatel Galego Roxo’ compared to the white-skinned variant, at both developmental stages. For the case of WRKY43 (VIT_14s0068g01770), despite an increased tendency in the red variant was observed, its differences were not statistically significant.

Our findings reveal a differential regulation of stilbene metabolism in color reverting berries. In grapevine, the overexpression of *MYBA* genes activates the initial steps of the phenylpropanoid pathway (activating genes such as *PAL* or *4CL*; Matus et al. [10]) despite they are not able to activate MYB14 nor MYB15 expression (note that these overexpression studies were conducted on grapevine hairy roots). Therefore, the stilbene pathway may be activated as a secondary response to the higher phenylpropanoid precursor availability. In addition, it may be also determined as a counterbalance mechanism to avoid the excessive accumulation of flavonoid compounds.

## Conclusions

In this study, we characterized the genetic, metabolic and transcriptomic differences caused by a white-*to*-red skin color reversion in a near-isogenic background, by comparing a red-skinned somatic variant and its white-skinned ancestor within ‘Moscatel Galego’ cultivars.

Several authors reported cyanidin derivatives as the minor group of anthocyanins in colored cultivars [43, 44]. Taking into account that cyanidin is the precursor of di-hydroxylated anthocyanins, it is usual to find low concentrations of its derivatives in red colored grapes [44]. Despite in black-skinned cultivars cyanidin derivatives constitute the minor group of anthocyanins, the data here obtained suggest that in both variants of cv. ‘Moscatel Galego’ the phenylpropanoid pathway is being majorly channeled into the di-hydroxylated flavonoid sub-branch (probably through the preferential activity of *F3’Ha*, *F3’H1*, *F3’H5*), resulting in a higher accumulation of cyanidin-3-*O*-glucoside in the red-skinned variant cv. ‘Moscatel Galego Roxo’ in contrast to the slight accumulation of tri-hydroxylated anthocyanins. Thus, we hypothesize that the main reason behind the pigmentation of the red-skinned variant is probably through a deficient activation of the tri-hydroxylated flavonoid sub-branch along with the decreased activity of anthocyanin-methylation/acylation enzymes.

The genetic data showed that the coloration of the red-skinned variant was recovered from the white-skinned phenotype of cv. ‘Moscatel Galego Branco’ by the partial activation of the *MYBA1* gene within the berry color locus. This conclusion was based on the fact that the red-skinned variants showed an heterozygotic partial excision of the *Gret1* retrotransposon from the promotor region of the anthocyanin regulator *MYBA1*, probably occurring exclusively in the L2 cell layer, while *MYBA2* was still in homozygosis for a non-functional allele. Altogether, these results allow to hypothesize a predictive model for the white-*to*-red color reversion in grapevine, specifically in cv. ‘Moscatel Galego’, where the partial *Gret1* excision from the promoter region of *MYBA1* (only in one allele), plus the lack of *MYBA2*’s function, lead to a decreased berry color locus activity and consequently to a decreased target activation that results in a mild-red skin instead of a blue/black-skinned coloration.

Regardless of our conclusions, the transcriptome differences found in the RNA-Seq analysis need to be interpreted with precaution as berry ripening progression differences between both cultivars may also be responsible for alterations in gene expression. Recent studies have shown that the color locus deletion is associated with other intra- and inter-chromosomal genomic rearrangements [7] and these other changes could impact the transcriptomic landscapes of somatic variants. Bud sports of cv. ‘Benitaka’ indeed show different sugar accumulation patterns [45]. ​​In tune with this data, cv. ‘Moscatel Galego Branco’ and its red-skinned variant, despite not showing differences in berry weight, pH and malic/tartaric acid accumulation, presented instead a difference in their sugar accumulation patterns [26], which could also influence other processes and their related gene expressions.

Finally, and probably as a consequence of the reactivation of the anthocyanin pathway in the red-skinned somatic variant, other related phenylpropanoid sub-branches may result affected. Such is the case of the stilbene pathway, a branch that is thought to compete against the flavonoid pathway despite we found it positively correlated to flavonoids in our analysis. Further studies are needed to demonstrate a direct effect of the anthocyanin pathway over the synthesis of stilbenes. Corroborating this observation, we previously observed a tendency in that resveratrol is lower in white skin cultivars compared to red and these in turn are lower compared to black-skinned individuals [25]. MYBA re-activation in addition to other unidentified factors could be responsible for increasing the flux into the stilbene pathway.

## Methods

### Plant material and geographical description of sampling sites

Young leaves, woody shoot and berries of *Vitis vinifera subsp. vinifera* cv. ‘Moscatel Galego Branco’ and cv. ‘Moscatel Galego Roxo’ were sampled from experimental vineyards planted in rows, located at Quinta de Santa Bárbara (near Pinhão) in Douro Region (41° 10′ N, 7° 33′ W, 130 m elevation; Additional file 8). Only healthy plants were sampled, presenting no visible signs of fungal attack or other microbiological alteration. In general, soils from this region have a high content of fine sand and silt and a low content of clay in the fine earth. They are acid and poor in organic matter [46]. The climatic regime is Mediterranean, with rains concentrated in winter and autumn, and a typical severe soil water deficit in summer. The average annual precipitation over 30 years is 650 mm. The maximum average precipitation generally occurs in winter (December, 100 mm approximately) while the minimum is in summer (August, 10 mm approximately). The mean annual air temperature is 16 °C and ranges from 8 °C in January, to 25 °C in July [46].

Fruit samples were collected around 10 a.m. in 2015 at two different developmental stages: 1) veraison, being characterized by the beginning of anthocyanin accumulation in cv. ‘Moscatel Galego Roxo’ (time point when 50% of berries have changed their color; at the same time cv. ‘ Moscatel Galego Branco’ was sampled); and 2) early ripening, corresponding to 2 weeks after veraison (WAV). On both sampling dates three independent biological replicates were randomly selected, each including 15–20 berries and belonging to a different group of 5–10 plants positioned on a same row within the vineyard. Berries were picked from both row sides and those collected at the veraison stage represent a pool of green-to-slightly pigmented berries in order to have the most reliable representation of this stage’s phenotype. Berries were immediately frozen in liquid nitrogen and kept at − 80 °C.

### Analysis of anthocyanin composition and content of berry skins

Anthocyanins were extracted from berry skins and quantified by high-performance liquid chromatography coupled to mass spectrometry (HPLC–MS), using standards for their identification, as recommended in García-Beneytez et al. [47]. All analyses were carried out using an HP 1100 system (Hewlett-Packard) with a PDA UV-vis coupled to a mass spectrometer equipped with an ESI interface. MS parameters were: capillary voltage, 4000 V; fragmenter ramped from 90 to 120 V; drying gas temperature, 325 °C; and gas flow (N_2_), 12 mL/min. The instrument was operated in positive ion mode scanning from *m/z* 50 to 2000 at a scan rate of 1.47 s/cycle.

A liquid chromatograph composed by a 600 quaternary pump, a 717 automatic injector, a TC2 controller for a column oven, a 996 photodiode array detector and a Millennium 32 workstation (Waters) was used to perform the HPLC analysis of anthocyanins. The separation was carried out using a Waters Nova-Pak C18 steel cartridge (3.9 × 250 mm), filled with 5-μm particles, and furnished with a Waters Sentry Nova-Pack C18 guard cartridge (20 × 3.9 mm), both thermostated at 55 °C. The two mobile solvent phases consisted of water/acetonitrile (95:5) adjusted to pH 1.3 with trifluoraectic acid (solvent A), and water/acetonitrile (50:50) adjusted to pH 1.3 with trifluoracetic acid (solvent B), using a gradient elution program as follows: linear gradient from 15% B to 35% B in 20 min, from 35% B to 50% B in 10 min, 50% B for 6 min, from 50% B to 100% B in 5 min, 100% B for 5 min, 100% B to 15% B in 1 min. A flow rate of 0.8 mL/min was applied. Samples (20 μL) were analyzed in triplicate. Spectra detection was registered every second between 250 and 600 nm, with a bandwidth of 1.2 nm. Samples, standard solutions, and mobile phases were filtered before analysis through a 0.45-μm pore size membrane.

### Genetic and transcriptomic analyses of cv. ‘Moscatel Galego’ white- and red-skinned variants

#### Single sequence repeat (SSR) analysis

Four genomic DNA samples were extracted for cv. ‘Moscatel Galego Branco’ and cv. ‘Moscatel Galego Roxo’ from 100 to 200 mg of young leaf and skin berry (L1 + L2 derived tissues) and from 200 to 300 mg of woody shoot pith and roots from greenhouse canes (L2 derived tissues) as previously described by Ferreira et al. [30].

Genomic DNA samples extracted from young leaves were analyzed for 12 Simple Sequence Repeat (SSR) loci - VVS2, VVMD5, VVMD7, VVMD27, VrZAG62, VrZAG79, VVMD28, VVMD32, VVIv67, VVIv37, VVIp31 and VMC4f3 in order to determine the genetic identity of both cv. ‘Moscatel Galego’ skin color variants. Polymerase chain reaction (PCR) conditions and further analysis were performed as described in Castro et al. [29]. Primer sequences are found in Additional file 9.

A set of 10 SSR markers (SC8_0146_010, SC8_0146_026, VVNTM1, VVNTM2, VVNTM3, VVNTM4, VVNMT5, VVNTM6, VVIU20, VMC7G3) flanking the berry color locus and distributed along the distal arm of chromosome 2 was used to investigate polymorphisms in this region, using genomic DNA samples, extracted from young leaf and woody shoot, taking advantage of a well-established layer-specific approach described by Vezzulli et al. [15]. For each marker, the genetic difference between the L1 + L2 (leaf) and L2 (woody shoot pith) should correspond to the make-up of the L1 cell layer. Primer sequences are found in Additional file 9. PCR reactions were performed as recommended in Vezzulli et al. [15]. Capillary electrophoresis was carried out in an ABI PRISM^R^ 310 Genetic Analyzer sequencer (Life Technologies) and the fragments were sized with Peak Scanner V1.0 software (Applied Biosystems) using as internal ladder the GeneScan 500 LIZ size standard (Life Technologies).

#### *MYBA1* and *MYBA2* gene structure analysis

Polymorphisms in *VvMYBA1* and *VvMYBA2* genes were determined as a proxy for the determination of functional and non-functional alleles. Genomic DNAs of young leaf, skin berry, woody shoot pith and roots were used to perform the *VvMYBA1* locus characterization. Primer pairs used for PCR analyses of *VvMYBA1* promoter region were VvMYBA1(1) for VvmybA1a allele detection (*Gret1* insertion) and VvMYBA1(2) for the detection of VvmybA1c and VvmybA1b alleles. Primer sequences are found in Additional file 9. The PCR reaction mixture (25 μL final volume) contained 50 ng total DNA, 0.3 mM dNTPs, 1 mM MgSO4, 0.3 μmol of each primer and 1 U Platinum™ *Pfx* DNA Polymerase (Life Technologies). The touch-down PCR was performed in a Veriti 96 well thermal cycler (Applied Biosystems) with the following steps: 5 min at 94 °C; 1 cycle at 94 °C for 15 s, 60 °C for 30 s, 68 °C for 2 min and 30 s; annealing temperature (Ta) decreased of 1 °C at each cycle up to 50 °C which was the Ta of the following 25 cycles; 68 °C for 5 min and a final step of at least 10 min at 4 °C to stop the reaction. Presence of PCR products was assessed by electrophoresis with a 1.5% agarose gel and quantified by comparison with NZYDNA Ladder III (NZYTech).

For *VvMYBA2* gene, the single nucleotide polymorphism (SNP) related to berry color VvMYBA2R44 (K980) was investigated by a SNaPshot assay. *VvMYBA2* amplification, SNP genotyping by ABI PRISM SNaPshot Multiplex kit (Life Technologies) and SNP detection on ABI PRISM 310 Genetic Analyzer Sequencer were performed as reported in Carrasco et al. [48].

#### RNA isolation

For gene expression studies (RT-qPCR and RNA deep sequencing), total RNA was extracted from berry skins according to the procedures described by Reid et al. [49]. To selectively precipitate the RNA, 125 μL of 10 M LiCl was added (final concentration 2.5 M in sample) and the samples were stored overnight at 4 °C. RNA was pelleted next day, washed with cold 70% EtOH, air dried and dissolved in 40 μL DEPC-treated water. Total RNA was further purified using the E.Z.N.A.® MicroElute RNA Clean Up Kit following the standard protocol. RNA concentration and 260/280 nm ratios were determined before and after DNase I digestion with a NanoDrop ND-1000 spectrophotometer (NanoDrop Technologies).

#### Transcriptome analyses

RNA-Seq analysis was performed on 12 samples corresponding to veraison (V) and early ripening (R, 2WAV) berries of cv. ‘Moscatel Galego Roxo’ (RV and RR) and cv. ‘Moscatel Galego Branco’ (WV and WR), with three biological replicates each. Pair-ended sequencing was performed on Illumina HiSeq2000 v4 with a sequencing lane of 2 × 50 bp and a depth of 40 million sequences approximately.

RNA-Seq data analysis was performed on the AIR platform (www.transcriptomics.cloud). More specifically, the quality of the raw reads was checked with FastQC (http://www.bioinformatics.babraham.ac.uk/projects/fastqc/) and then a trimming step was performed to remove low quality bases and sequencing adaptors with the tool BBDuk (https://sourceforge.net/projects/bbmap/). A minimum Phred-like score of 25 was set and a minimum length of the reads of 25 nucleotides. High quality reads were then mapped on the reference genome (IGPP 12Xv1) of *Vitis vinifera* cv. ‘Pinot Noir’ PN40024 with STAR (https://github.com/alexdobin/STAR) considering Pinot and Moscatel as different genotypes. The STAR parameters were the following: maxMismatch: 5; maxMismatchRatio: 0.3; minScoreRatio: 0.66; alignEndsType Local. Read summarization was then performed with featureCounts (http://bioinf.wehi.edu.au/featureCounts/) using only the reads with a mapping quality higher than 30. The statistical analysis was performed with R. Specifically, lowly expressed genes were removed with the package HTSFilter, a method that automatically calculates the minimum level of expression (TMM) for a gene to pass into the statistical analysis and to optimize the similarity of replicates (http://www.bioconductor.org/packages/release/bioc/html/HTSFilter.html), selecting “TMM” as the normalization method. PCA was performed on the TMM normalized gene matrix after the removal of the not expressed genes, using the R function prcomp. Then, the filtered genes were used to perform a differential analysis with DESeq2. Genes were considered statistically differentially expressed if the corrected *p*-value (false discovery rate, FDR) was lower than 0.05 (Additional file 10). Gene Ontology Enrichment Analysis (GOEA) was performed with in house scripts based on a hypergeometric test on the proportion of GO categories between the differentially expressed genes (DEGs) and the whole genome, GO categories were considered enriched if the FDR of the test was less than 0.05. The original sequence data were submitted to the Sequence Read Archive (SRA) database of the NCBI under the accession SRP156198 (BioProject ID PRJNA484195).

A complementary RNA-Seq analysis was performed by adding publicly available data from the dark-skinned table grape cv. ‘Red Globe’ and its purple-*to*-red color somatic variant cv. ‘Chimenti Globe’ (SRA BioProject PRJNA539972, [50]). Additionally, when the flavonoid 3′ and 3′5’ hydroxylase families were studied, Fragments per kilobase of transcript per million mapped fragments (FPKMs) were calculated from both multi-mapped and filtered reads for further comparisons (Additional file 11).

Expression heatmaps were constructed for different list of genes of interest (corresponding to different branches of the phenylpropanoid pathway) using the web-based tools Heatmapper (http://www3.heatmapper.ca/expression/) or ClustVis (https://biit.cs.ut.ee/clustvis/), using log_2_FPKM + 1 or transforming FPKM values using a Z-score scaling whenever needed.

#### Quantitative reverse transcription PCR (RT-qPCR) expression analysis

Total RNA (200 ng) was reverse transcribed with the qScript™ cDNA SuperMix (Quanta Biosciences) according to the manufacturer’s guideline. The differential transcript level of *MybA1/2*, *UFGT*, *F3’H1a* and *F3’5’H1a* was determined by RT-qPCR using a 7300 Real Time PCR System (Applied Biosystems). Each reaction (20 μL) contained 200 nM of each primer, 3 μL of cDNA (1:30 dilution of the synthesis reaction), 1x SYBR Green Real-Time PCR Master Mix (Thermo Fisher) and water up to 20 μL. Thermal Cycling conditions were 95 °C for 10 min, followed by 95 °C for 15 s, annealing (variable temperatures) for 30 s and 72 °C for 30 s for 40 cycles. A melting cycle with temperature ranging from 60 to 95 °C was included in order to detect non-specific amplification in cDNA samples. Each one of the three biological replicates used to extract RNA and synthetize cDNA were used for RT-qPCR reaction in two technical replicates. Gene transcripts were quantified upon normalization to Elongation factor 1-alpha (*EF1α*, VIT_06s0004g03220) by comparing the threshold cycle (Ct) of each target gene with geometric mean of *EF1α* Ct. The relative quantification per each gene was calculated by the 2^-ΔCt^ method, where ΔCt is the difference in threshold cycle between the geometric means of the target gene and the reference gene.

#### Stilbene quantifications

Extraction of total stilbenes from berry skins was conducted as described by Vrhovsek et al. [51]. The quantification of piceid and resveratrol was performed by injecting 10 μl of extract in an Agilent 1100 Series HPLC coupled with an Agilent Technologies 6120 Quadrupole LC/MS. Reverse phase separation was carried out in a column Synergi 4u Fusion-RP 80, 150 × 4.6 mm 4 μm Phenomenex, flow rate was 0.5 mL/min using gradient Solvent A (1% formic acid in water) and solvent B (1% formic acid in acetonitrile). The solvent gradient was as follows: 0–20 min from 90 to 40% solvent A; 20–21 min from 40 to 10% solvent A; 21–26 min hold 10% solvent A; 26–27 min from 10 to 90% solvent A; 27–37 min hold 90% solvent A. The compounds were determined and quantified by comparing with trans-resveratrol (R5010 Sigma-Aldrich, St. Louis) and piceid (15,721 Sigma-Aldrich, St. Louis) standards.

## Supplementary information

**Additional file 1.** A) Principal component analysis (PCA) of red and white-skinned somatic variant transcriptomic data sets, performed on the TMM normalized gene matrix after the removal of the not expressed genes. B) Number of differentially expressed genes in each comparison.

**Additional file 2.** Expression values (FPKM) calculated for all grapevine genes (30 K genes) for each biological replicate. FDR values from the DEA are included as separate sheets.

**Additional file 3.** Gene Ontology Enrichment Analysis in up- and down-regulated genes (DESeq2) within RV vs WV comparison (FDR = < 0,05; Query Item> = 3).

**Additional file 4.** Gene Ontology Enrichment Analysis in up- and down-regulated genes (DESeq2) within RR vs WR comparison (FDR = < 0,05; Query Item> = 3).

**Additional file 5.** Common and unique gene ontology terms in up- and down-regulated genes at both veraison and ripening stages.

**Additional file 6.** Phenylpropanoid pathway genes with normalized and averaged expression values (FPKM) obtained from this study. R: red-skinned variant (cv. ‘Moscatel Galego Roxo’), W: white-skinned variant (cv. ‘Moscatel Galego Branco’), V: veraison, R: ripening (2WAV).

**Additional file 7.** Gene expression behavior of anthocyanin-branch genes in skin color somatic variants of cv. ‘Moscatel Galego’ and cv. ‘Red Globe’. A) Heatmap of logFPKM+ 1 transformed data produced using anthocyanin gene expressions obtained in the ‘Moscatel’-‘Red Globe’ RNA-Seq integrative analysis. Genes (rows) are clustered using correlation distance and average linkage. B) Principal component analysis (PCA) produced from logFPKM+ 1 values of anthocyanin structural genes.

**Additional file 8.** Geographic location of cv. ‘Moscatel Galego’ individuals used in this study; cv. ‘MGB Moscatel Galego Branco’ (MGB) and its somatic variant cv. ‘MGR’. The figure was produced by satellite pictures made available in Google Earth.

**Additional file 9.** List of primers used for molecular analyses.

**Additional file 10.** Volcano plots of gene expression changes in the four comparisons tested.

**Additional file 11. **FPKM values of the *F3’5’H* and *F3’H* gene families in cv. ‘Moscatel Galego’ and cv. ‘Red Globe’ skin color somatic variants. FPKM values were calculated from A) multiple-mapped and B) filtered reads and are displayed for each biological replicate.

## Data Availability

SRA accession SRP156198. BioProject ID PRJNA484195.

## Abbreviations

3AT: Anthocyanin 3-*O-*glucoside-6″-*O-* acyltransferase
4CL: 4-coumarate: CoA ligase
AOMT: Anthocyanin *O-*methyltransferase
bp: Base pair
C4H: Cinnamate-4-hydroxylase
cDNA: Complementary deoxyribonucleic acid
Ct: Threshold cycle
DEGs: Differentially expressed genes
EF1α: Elongation factor 1 alpha
F3’5’H: Flavonoid 3′,5′-hydroxylase
F3’H: Flavonoid 3′-hydroxylase
FDR: False discovery rate
FPKM: Fragments per kilobase of transcript per million mapped fragments
GO: Gene ontology
GOEA: Gene ontology enrichment analysis
*Gret1*: Grape retrotransposon 1
GST: Glutathione *S*-transferase
HPLC-MS: High-performance liquid chromatography coupled to mass spectrometry
INDEL: Insertion or deletion
LTR: Long terminal repeat
mRNA: Messenger ribonucleic acid
MYB: myeloblastosis
OMT: *O*-methyltransferase
PAL: Phenylalanine ammonia lyase
PC: Principal component
PCA: Principal component analysis
PCR: Polymerase chain reaction
PP: Phenylpropanoid pathway
RT-qPCR: Quantitative reverse transcription polymerase chain reaction
siRNA: small interference RNA
SNP: Single-nucleotide polymosphism
SNV: Single nucleotide variation
SSR: Simple sequence repeat
STS: Stilbene synthase
SV: Structural variation
TF: Transcription factor
UDP: Uridine diphosphate
UFGT: UDP-glucose: flavonoid 3-*O*-glucosyltransferase
UTR: Untranslated region
WAV: Weeks after veraison
WRKY: Tryptophan-arginine-lysine-tyrosine (WRKY) protein domain
