# Supplementary figures and images for: Genetic analysis of a white-to-red berry skin color reversion and its transcriptomic and metabolic consequences in grapevine (Vitis vinifera cv. ‘Moscatel Galego’)
Source: BMC Genomics. 2019 Dec 9;20:952. doi: 10.1186/s12864-019-6237-5 (PMC6902604; doi:10.1186/s12864-019-6237-5)

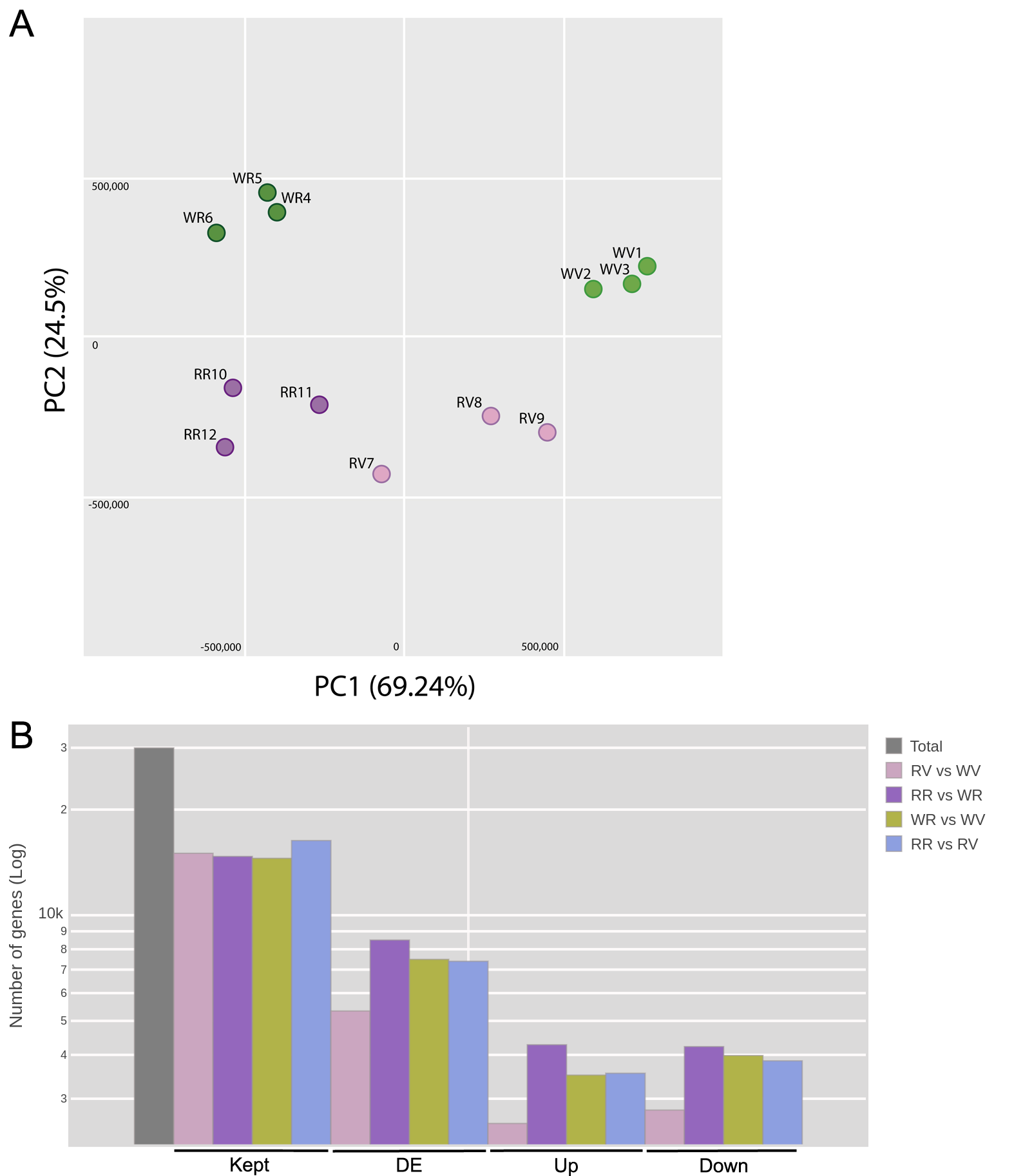

Supplement: Supplementary file 1 — Additional file 1. A) Principal component analysis (PCA) of red and white-skinned somatic variant transcriptomic data sets, performed on the TMM normalized gene matrix after the removal of the not expressed genes. B) Number of differentially expressed genes in each comparison. [file 12864_2019_6237_MOESM1_ESM.png]

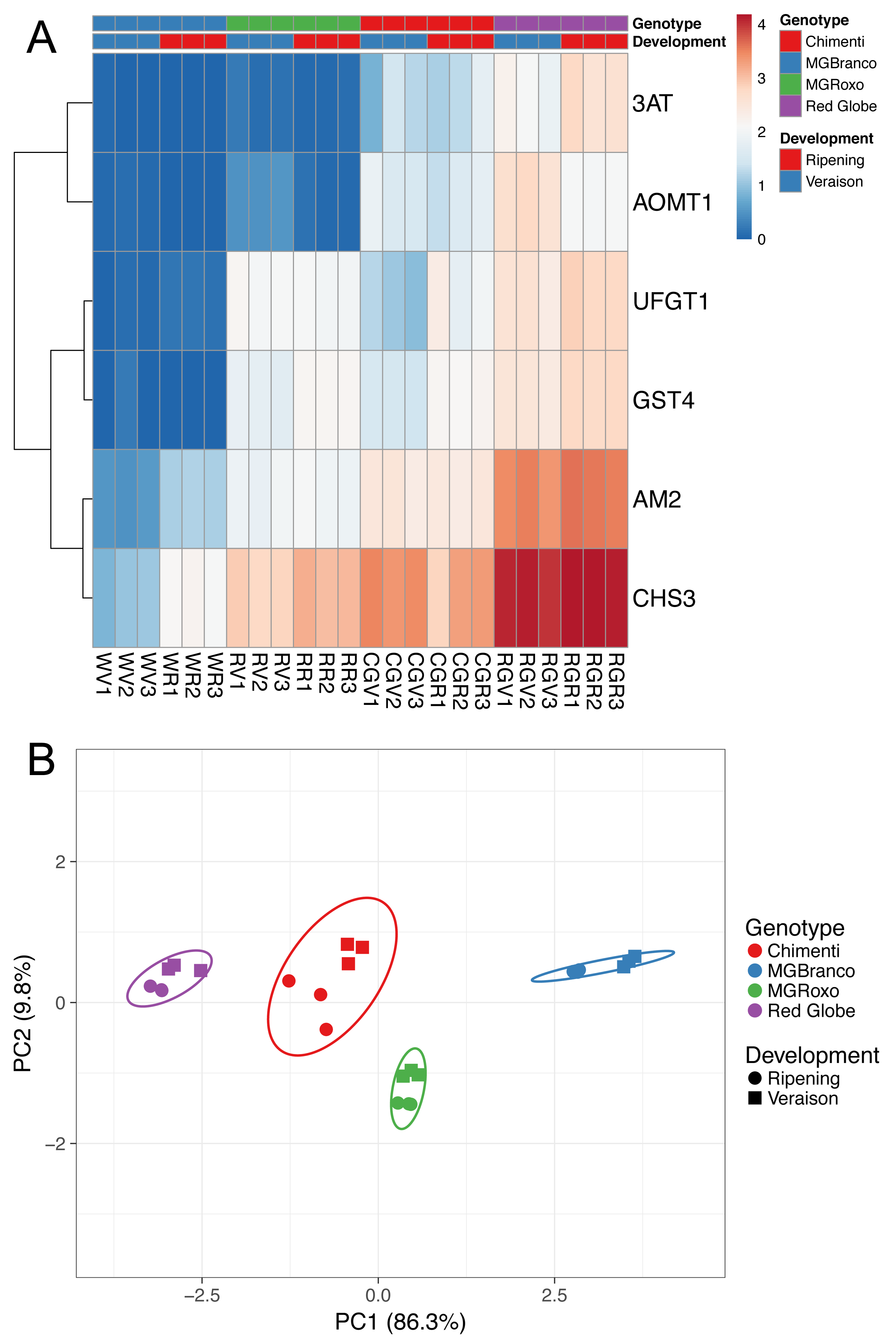

Supplement: Supplementary file 7 — Additional file 7. Gene expression behavior of anthocyanin-branch genes in skin color somatic variants of cv. ‘Moscatel Galego’ and cv. ‘Red Globe’. A) Heatmap of logFPKM+ 1 transformed data produced using anthocyanin gene expressions obtained in the ‘Moscatel’-‘Red Globe’ RNA-Seq integrative analysis. Genes (rows) are clustered using correlation distance and average linkage. B) Principal component analysis (PCA) produced from logFPKM+ 1 values of anthocyanin structural genes. [file 12864_2019_6237_MOESM7_ESM.png]

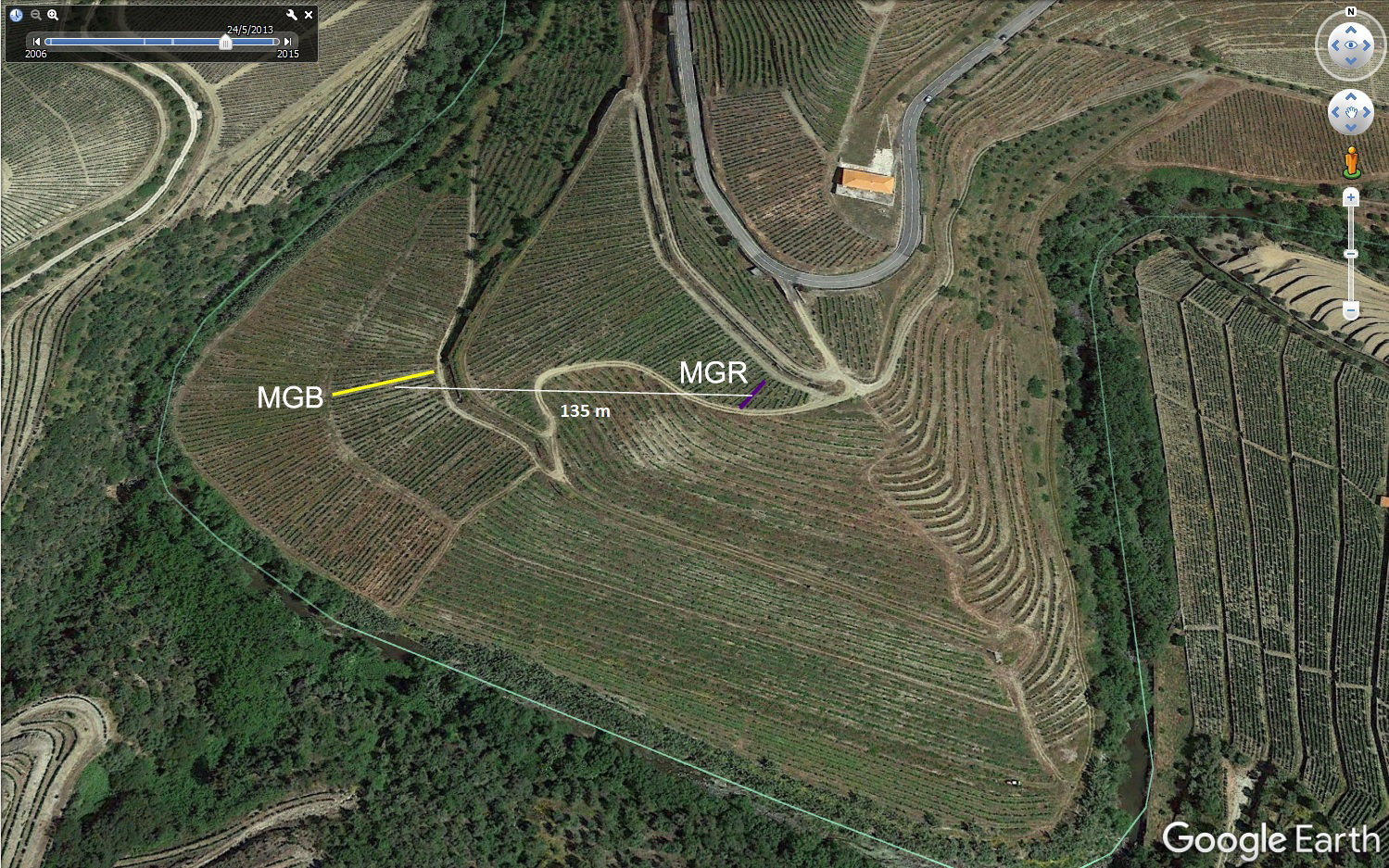

Supplement: Supplementary file 8 — Additional file 8. Geographic location of cv. ‘Moscatel Galego’ individuals used in this study; cv. ‘MGB Moscatel Galego Branco’ (MGB) and its somatic variant cv. ‘MGR’. The figure was produced by satellite pictures made available in Google Earth. [file 12864_2019_6237_MOESM8_ESM.png]

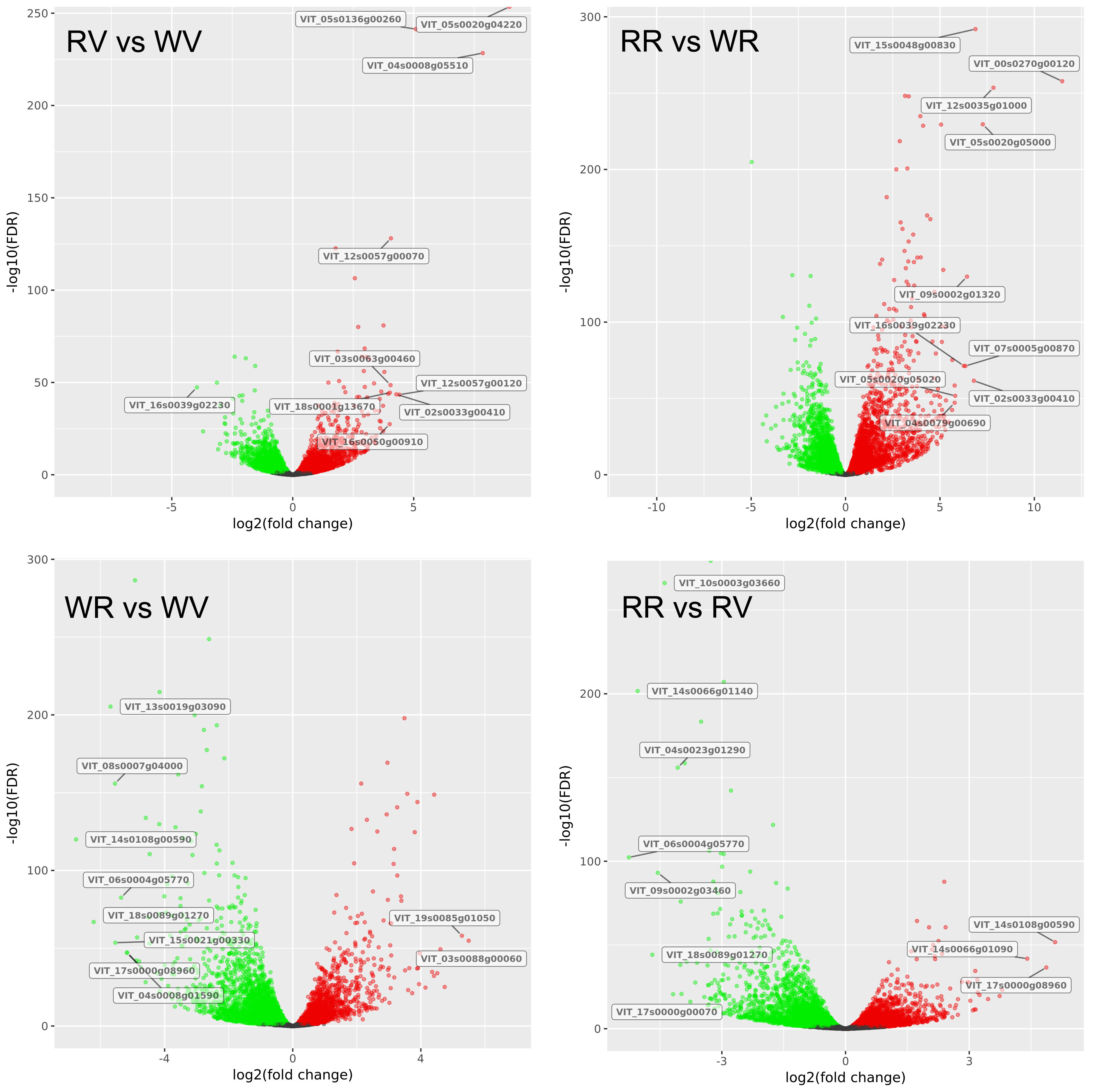

Supplement: Supplementary file 10 — Additional file 10. Volcano plots of gene expression changes in the four comparisons tested. [file 12864_2019_6237_MOESM10_ESM.png]
